# Deciphering The Potential Role of Hox Genes in Pancreatic Cancer

**DOI:** 10.3390/cancers11050734

**Published:** 2019-05-27

**Authors:** Tzu-Lei Kuo, Kuang-Hung Cheng, Li-Tzong Chen, Wen-Chun Hung

**Affiliations:** 1 National Institute of Cancer Research, National Health Research Institutes, Tainan 704, Taiwan; rayfish@nhri.org.tw (T.-L.K.); leochen@nhri.org.tw (L.-T.C.); 2 Institute of Biomedical Sciences, National Sun Yat-Sen University, Kaohsiung 804, Taiwan; khcheng@faculty.nsysu.edu.tw; 3 Division of Hematology/Oncology, Department of Internal Medicine, National Cheng Kung University Hospital, Tainan 704, Taiwan; 4 Graduate Institute of Medicine, College of Medicine, Kaohsiung Medical University, Kaohsiung 807, Taiwan

**Keywords:** Hox genes, pancreas development, pancreatic cancer, genetically engineered mouse

## Abstract

The Hox gene family plays an important role in organogenesis and animal development. Currently, 39 Hox genes that are clustered in four chromosome regions have been identified in humans. Emerging evidence suggests that Hox genes are involved in the development of the pancreas. However, the expression of Hox genes in pancreatic tumor tissues has been investigated in only a few studies. In addition, whether specific Hox genes can promote or suppress cancer metastasis is not clear. In this article, we first review the recent progress in studies on the role of Hox genes in pancreatic cancer. By comparing the expression profiles of pancreatic cancer cells isolated from genetically engineered mice established in our laboratory with three different proliferative and metastatic abilities, we identified novel Hox genes that exhibited tumor-promoting activity in pancreatic cancer. Finally, a potential oncogenic mechanism of the Hox genes was hypothesized.

## 1. Introduction

During development, the maintenance of cellular identity and proper segmentation are essential for the generation of normal tissue structure and organ function in embryos and adult animals. One of the gene families that participate in the control of organogenesis and development is Hox. This name comes from the fact that proteins encoded by this gene family contain a functional homeobox (Hox) domain, with a length of around 60 amino acids that exhibits DNA-binding activity [1].

## 2. Important Features of Hox Genes

Hox genes were initially discovered as a group of transcriptional regulators that played a critical role in embryogenesis and body segmentation in Drosophila [1]. Mutations in Hox genes cause alterations of body structure like the replacement of antennae to legs or a lack of wings [2]. In Drosophila, there are eight Hox genes that aggregate into two genomic clusters, both on chromosome 3 [3,4]. By the end of the 1980s, two research groups identified Hox genes in the mouse genome. Molecular characterization revealed similarities in the genomic organization and gene expression patterns of these genes in mice and Drosophila [5,6]. To date, emerging evidence shows that the Hox genes are highly conserved in animals including horn shark, tilapia, zebrafish, striped bass, mice, and humans [7,8,9].

In humans, a total of 39 Hox genes have been cloned and split into four clusters: HoxA, HoxB, HoxC, and HoxD. These clusters are located on chromosomes 7, 17, 12, and 2, respectively [10]. One crucial feature in the transcriptional regulation of Hox genes is the order (or consequence) in which individual genes are expressed sequentially in different embryonic regions in a head-to-tail manner to ensure correct segmentation [11]. Another important feature is the linear organization of Hox genes in clusters. The earlier-expressed Hox genes are located at the 3′-end of the cluster, while the late-expressed genes are located at the 5′-end [12]. 

How the expression of Hox genes is coordinately controlled is an intensively investigated issue. At least three regulatory mechanisms have been proposed to contribute to the complex control of Hox gene transcription. First, the expression of late Hox genes can be activated by products encoded by the earlier-expressed Hox genes [13]. Second, posterior genes located near the 5′-end of the cluster may suppress the expression of anterior Hox genes via the recruitment of the Polycomb repression complex [14,15]. Finally, crosstalk between various Hox genes may establish a delicate network to regulate sequential expression during development [16,17]. 

## 3. Involvement of The Hox Genes in Pancreas Development

The pancreas is a bi-functional gland with both exocrine and endocrine activities. As an endocrine gland, it plays an important role in regulating the blood sugar level by secreting insulin, glucagon, and somatostatin. As an exocrine gland, it secretes pancreatic enzymes into the duodenum through the pancreatic duct to help with the digestion of fats, proteins, and carbohydrates. 

The pancreas develops from endodermal cells in the future midgut region of embryos. The dorsal pancreatic primordium is initiated in the endoderm by signaling that is induced by the secreted proteins, activin, and basic fibroblast growth factor, released from the overlying notochord. The expression pattern of Hox genes in the early endoderm indicates that HoxA4, HoxA5, and HoxB4 provide the spatial information needed to limit the response to signals from the notochord [11,12,13]. Mouse Hox6 is expressed exclusively in the mesoderm of the developing pancreas. Genetic knockout of all three Hox6 paralogs (HoxA6, HoxB6, and HoxC6) causes a dramatic loss of endoderm-derived endocrine cells, including β-cells, and a disruption in pancreatic branching and exocrine differentiation [14]. The expression of another member of this family, HoxD1, correlates with the expression of glucagon-like peptide-1 and glucose-dependent insulinotropic peptide that participate in stimulating hormone secretion [15]. Furthermore, retinoic acid, which regulates Hox expression by binding to nuclear hormone receptors, also plays a key role in pancreas development [16]. 

Several Hox genes—including HoxA4, HoxA5, HoxB4, and HoxA11—are activated directly by retinoic acid, and increases in expression of these genes are sufficient to drive embryonic stem cell differentiation toward functional insulin producing cells [17]. HoxA1 is expressed in pancreatic cell populations as two alternatively spliced mRNAs. The encoded proteins share their N-terminal domain, but either lack or include the homeobox domain at the C-terminus. HoxA1 is an early response gene for transforming growth factor-ß in pancreatic epithelial cell populations, and the HoxA1 protein is co-localized with transforming growth factor-ß receptors in the embryonic pancreatic epithelium at the time when exocrine pancreatic morphogenesis occurs [18]. In addition to the Hox genes, the paired box (Pax) genes, characterized by the presence of a pair of partial or complete homeodomains, are also crucial transcriptional co-regulators that control α- and β-cell development [19,20]. 

The Pax and Hox binding sites are generally located at the distal promoter region of target genes. These two family members can occupy their consensus sites separately to interact and work together in order to activate gene transcription [21]. Pax genes may act as upstream regulators of Hox genes. For example, Pax6 directly controls HoxD4 enhancer activity, indicating that Pax genes cross-regulate Hox genes [22]. The aforementioned results suggest that Hox genes are indispensable for pancreatic development.

## 4. Dysregulation of Hox Gene Expression in Pancreatic Cancer

Several studies have indicated that Hox proteins may play a key role in the development of cancers. Aberrant expression of Hox genes has been reported in colorectal cancer [23], breast cancer [24], prostate cancer [25], lung cancer [26], glioblastomas [27], ovarian cancers [28], and leukemias [29]. One notable member is HoxB7, which increases invasive ability in breast [30], lung [31], ovarian [32], and pancreatic [33] cancers. When HoxB7 was depleted by small interfering RNA, migration of Panc1, BxPC-3, and Miacapa2 pancreatic cancer cells was suppressed, while proliferation and cell viability were unaffected. In a clinical setting, the HoxB7 protein level correlated with lymph node metastasis [33]. 

In the HoxB gene cluster, several members including HoxB2, HoxB5, HoxB6, and HoxB13 are over-expressed in pancreatic cancer [34,35,36]. HoxB13 expression is significantly higher in tumor (68.2%) than in adjacent non-tumor tissue (22.4%). Moreover, high HoxB13 correlates with tumor angiogenesis and aggressive clinicopathological characteristics, and could serve as a promising marker for unfavorable prognosis in pancreatic cancer [35]. 

Members of the HoxA cluster are also upregulated in pancreatic cancer. For example, HoxA5, which is highly expressed in the adult and embryonic foregut but not in the normal adult pancreas, is frequently increased in early pancreatic intraepithelial neoplasia (PanIN) lesions when compared to normal ductal epithelium. Interestingly, urokinase plasminogen activator may act via the HoxA5 protein to promote cancer stemness [37]. A role for HoxA9 in the self-renewal of cancer cells has been suggested in pancreatic cancer. The long non-coding RNA, HOTTIP, binds with WD repeat-containing protein 5 to enhance HoxA9 expression, which, in turn, activates the Wnt/β-catenin pathway in pancreatic cancer cells to promote stemness properties. Knockdown of HoxA9 impairs sphere formation and downregulates the expression of stem factors including Lin28, Nanog, Oct4, and Sox2 [38]. On the other hand, another member of the Hox family, HoxA13, regulates HOTTIP to enhance cancer progression and gemcitabine resistance. Down-regulation of the HoxA13 protein reduces proliferation, invasion, and chemoresistance of pancreatic cancer cells. Immunohistochemical staining revealed that higher HoxA13 expression was correlated with lymph node metastasis, poor histological differentiation, and decreased overall survival in pancreatic cancer patients [39].

Several HoxD genes, such as HoxD13, are upregulated in breast cancer, melanoma, cervical cancer, and astrocytomas [40,41,42,43]. Significant differences in expression are detectable between normal (16.1%) and cancerous (57.7%) tissues, with the majority of tumors showing increased HoxD13 expression. Results of these studies suggest an oncogenic role of HoxD13 in the tumors. In contrast, HoxD13 seems to function as a tumor suppressor in pancreatic cancer [44]. In micro-dissected pancreatic tumors, HoxA1 protein is overexpressed in pancreatic cancer and negatively correlates with miR-10a. However, inhibition of HoxA1 promotes the invasiveness of pancreatic cancer cells [45]. A possible explanation is that HoxA1 is upregulated at the early stage of carcinogenesis, and then downregulated at the more invasive stage—suggesting a change of biological function during cancer initiation and progression. 

In this article, we mainly discussed the alterations of Hox genes in pancreatic cancer, because the mutation rate of Hox genes in human pancreatic tumor tissues is very low (<1%). Currently, only HoxD4 and HoxB13 have been shown to be mutation targets in limited cancers. Therefore, the contribution of Hox gene mutation in pancreatic tumorigenesis awaits further investigation. Another critical issue to be answered is the functional redundancy of the Hox gene members. Under various physiological or pathological circumstances, the increase or decrease of a specific member may lead to compensatory expression of other members. It is also possible that the switch of expression from one member to another member may elicit a compensatory oncogenic or tumor-suppressive role of the Hox genes. Because there are 39 Hox genes identified in humans, the complexity of the regulation of Hox gene members is very high. More sophistical approaches like the animal models described below are needed to clarify the functional crosstalk of the Hox gene members.

## 5. Novel Hox Genes in Pancreatic Tumorigenesis Identified by Genetically Engineered Mouse (GEM) Models

GEM models are useful for understanding pathological changes during tumorigenesis, and provide important information for the development of targeted therapies. Several GEM models of pancreatic cancer have been established by deleting tumor suppressor genes and introducing mutated genes into mice to mimic the genetic alterations found in primary pancreatic tumors. The most highly mutated gene in pancreatic cancer is Kras, which occurs in over 90% of human pancreatic tumors [46,47]. The second most prevalent genetic defect in pancreatic cancer is p53 inactivation via mutation, deletion, or promoter methylation. Other frequently inactivated genes include cyclin-dependent kinase inhibitor 2A, SMAD4, and AT-rich interactive domain-containing protein 1A [48]. Mutated Kras induces PanIN formation and cooperates with the inactivation of tumor suppressor genes to promote the progression of PanIN to pancreatic cancer or advanced pancreatic cancer [48,49,50].

**Figure 1:**
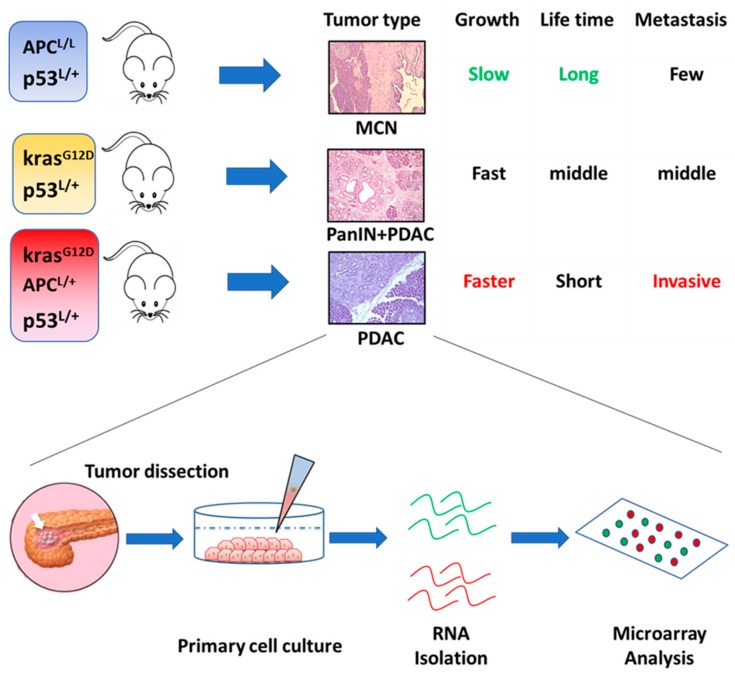
The three genetically engineered mouse models of pancreatic cancer established in our laboratory, and a flowchart for the study of gene expression profiles.

Recently, we created three GEM models of pancreatic cancer (Figure 1). The first model was of PA53 mice, which exhibited a heterozygous loss of the adenomatous polyposis coli gene coupled with p53 deficiency. These mice develop a mucinous cystic neoplasm at an early stage, which slowly progresses into carcinoma [51]. The second model was the classic KPC mice, which exhibit a Kras mutation and p53 inactivation. These mice develop PanIN around 8–10 weeks of age, and malignant tumors about 12 weeks after birth. The third model of KPA mice was the most aggressive. These mice contain all three genetic defects mentioned in the previous two GEM models [52]. We compared the gene expression profiles of pancreatic cancer cells isolated from PA53 and KPC mice by microarray analysis. Our results identified seven upregulated and three downregulated Hox genes in KPC relative to PA53 mice (Table 1). 

When we compared the expression of Hox genes in KPA and KPC mice, we found four upregulated and three downregulated Hox genes in the most aggressive KPA tumors (Table 2).

**Figure 2:**
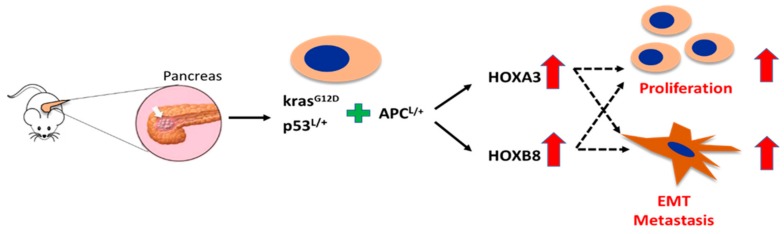
Schematic illustrating the hypothetical roles for HoxA3 and HoxB8 in pancreatic cancer proliferation and metastasis.

Interestingly, the expression of HoxA3 and HoxB8 was continuously upregulated in pancreatic cancer cells with increased metastatic ability, while the expression of HoxA10, HoxA11, and HoxB13 was consistently reduced in these cells. Our results suggest that these novel Hox genes may be involved in the malignancy or metastasis of pancreatic cancer. A previous study demonstrated that HoxA3 promoted tumor growth and metastasis in colon cancer [53], but the biological function of HoxA3 in pancreatic cancer has never been addressed. Very recently, HoxA3 has been shown to be a key mediator of an oncogenic long non-coding RNA, HoxA-AS2 [54]. As HoxA3 is significantly increased in KPA cells that exhibit aggressive behavior, we believe that HoxA3 acts as an oncogene in pancreatic cancer (Figure 2). 

As with HoxA3, no study has previously investigated the expression and clinical significance of HoxB8 in pancreatic cancer. Upregulation of HoxB8 was first described in human colon cancer [55]. Subsequently, this gene was found to be overexpressed in leukemia cells and to inhibit the differentiation of myeloid cells [56]. Recently, HoxB8 was shown to promote the epithelial-to-mesenchymal transition and metastasis in gastric and colon cancers [57,58]. We hypothesize that HoxB8 is also a metastasis promoter in pancreatic cancer. Downregulation of HoxA10, HoxA11, and HoxB13 in KPA cells is unexpected, because these Hox genes act as tumor-promoting genes in several types of cancers [59,60,61,62]. There are two possible explanations for this discrepancy. First, the oncogenic activity of these three Hox genes may be cell context-dependent. For example, HoxA10 increases p53 expression and reduces matrix invasion in breast cancer cells, indicating a tumor suppressor function [63]. However, this gene has been suggested as an oncogene in various cancers [64,65,66]. Second, the comparison of gene expression was done in isolated pancreatic cancer cells that lacked stromal cells. In vivo, the behavior and gene expression profile of cancer cells is affected by the tumor microenvironment. Therefore, it is possible that the in vitro expression of HoxA10, HoxA11, and HoxB13 differs from that of tumor tissues in vivo. 

## 6. Clinical Significance of Hox Gene Dysregulation in Pancreatic Cancer

**Figure 3:**
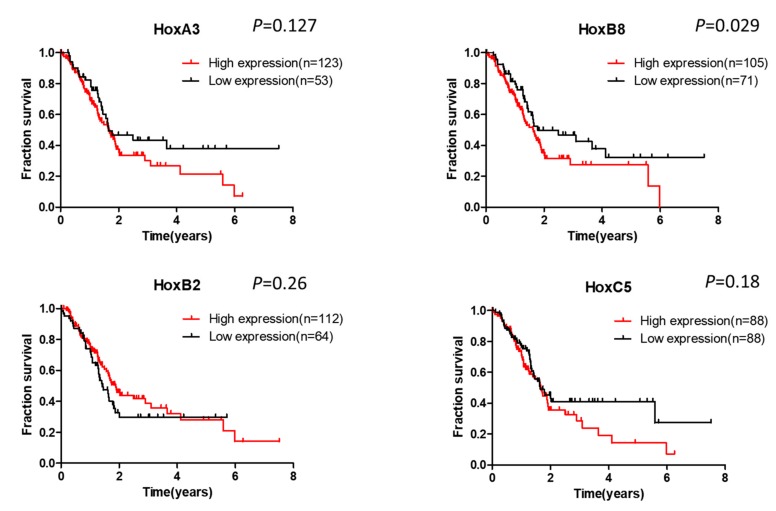
The association of expression of Hox genes with the survival of pancreatic cancer patients. Data were collected from the public database The Human Protein Atlas.

Until now, few studies reported the association of Hox expression with clinicopathological features in pancreatic cancer. We searched the information from a public database The Human Protein Atlas (https://www.proteinatlas.org). As shown in Figure 3, high expression of HoxA3, B8, and C5 is correlated with poor survival in pancreatic cancer, suggesting an oncogenic role of these members. On the contrary, HoxB2 expression is not linked with survival. The result shown in the database is the expression of mRNA. The protein level of Hox members in pancreatic cancer and their association with clinical outcome needs further characterization.

## 7. Conclusions and Future Perspectives

Pancreatic cancer is a deadly disease with a five-year survival rate of less than 8%. Currently, gemcitabine-based chemotherapy is the first line treatment for this cancer. However, the response is not satisfactory. Therefore, the identification of novel genes involved in cancer initiation, promotion, and progression is important for the development of new therapeutic strategies. Although some evidence suggests the involvement of Hox genes in pancreatic cancer, our understanding of the functions of different members of this gene family in pancreatic cancer is still at a preliminary stage. By using a global approach, future studies may reveal the role(s) of the novel Hox genes identified in our study. Moreover, the results of our study may benefit pancreatic cancer patients by directing the development of newly targeted therapies. 

## Figures and Tables

**Table 1:** Differential expression of Hox genes in KPC and PA53 mice.

	KPC/PA53	
Gene Name	Fold Change (log2)	*p*-Value
HoxB2	3.508	<0.001
HoxC5	2.940	0.007
HoxB8	2.402	<0.001
HoxA3	2.185	<0.001
HoxB9	2.098	<0.001
HoxA1	1.867	<0.001
HoxB3	1.249	0.038
HoxB13	−2.188	0.028
HoxA10	−2.605	<0.001
HoxA11	−4.501	<0.001

**Table 2:** Differential expression of Hox genes in KPA and KPC mice.

	KPA/KPC	
Gene Name	Fold Change (log2)	*p*-Value
HoxA3	5.297	<0.001
HoxB4	1.939	<0.001
HoxB8	1.421	<0.001
HoxA2	1.077	0.031
HoxA11	−1.278	0.014
HoxA10	−2.519	<0.001
HoxB13	−3.178	<0.001
